# Evidence‐Based Mouth and Oral Health Individualised Treatment Plan in Adults With Acquired Brain Injury: A Cluster Nonrandomized Controlled Trial

**DOI:** 10.1111/joor.70170

**Published:** 2026-02-16

**Authors:** Mohit Kothari, Silas Alves‐Costa, Gustavo G. Nascimento, Daniel M. Belstrøm, Lena Aadal, Jørgen Feldbæk Nielsen, Simple F. Kothari

**Affiliations:** ^1^ Hammel Neurorehabilitation Centre and University Research Clinic, Department of Clinical Medicine Aarhus University Hammel Denmark; ^2^ Research Unit of Dentistry, Department of Clinical Research, Faculty of Health Sciences University of Southern Denmark Odense Denmark; ^3^ Dentistry Graduate Program, Federal University of Maranhão São Luís Brazil; ^4^ School of Dentistry, University of Utah Salt Lake City Utah USA; ^5^ Section for Clinical Oral Microbiology, Department of Odontology University of Copenhagen Copenhagen Denmark; ^6^ Department of Clinical Neurophysiology, Aarhus University Hospital & Department of Clinical Medicine Aarhus University Aarhus Denmark

**Keywords:** acquired brain injury, bleeding on probing, individualised oral care, multidisciplinary care, neurorehabilitation

## Abstract

**Objective:**

Oral health care (OHC), despite its essential role in overall health, remains underprioritized in medical practice. The study investigated the effectiveness of an individualised, item‐specific, Mouth and Oral Health Individualised Treatment (MOHIT) plan compared to the existing oral care plan (EOCP) in individuals with acquired brain injury (ABI).

**Methods:**

A cluster nonrandomized controlled trial was conducted across six wards at a neurorehabilitation center. Wards were pragmatically assigned to deliver either MOHIT or routine EOCP for 3 weeks. Based on stepwise oral assessment, the MOHIT plan provided individualised protocols tailored to each patient's dependency level and oral condition. Oral care was performed daily by trained nurses under dental supervision. The primary outcome was change in bleeding on probing (BOP) from baseline to Week 4. Secondary outcomes included plaque index, tongue coating, oral malodor, salivary flow rate, and modified Bedside Oral Examination (mBOE) total and sub‐scores. Linear mixed‐effects models (for continuous outcomes) and cumulative link mixed models (for ordinal outcomes) were applied to assess Week‐4 versus baseline changes between groups, adjusting for ward clustering, baseline values, age, sex, and feeding status.

**Results:**

86/126 individuals completed the trial (34 MOHIT, 52 EOCP). Compared with EOCP, the MOHIT plan showed greater reductions in BOP (coeff: −19.11; 95% CI: −27.97 to −10.24) and significant improvements in plaque (−13.23; 95% CI: −19.93 to −6.53), tongue coating (−3.01; 95% CI: −5.58 to −0.45), oral malodor (−1.27; 95% CI: −2.15 to −0.40), and suspected oral candidiasis (−3.21; 95% CI: −3.22 to −3.20) compared with EOCP. MOHIT also showed greater improvement in mBOE scores (−1.46; 95% CI: −2.54 to −0.39). Effects remained consistent after sensitivity analyses controlling for baseline imbalance and missing data.

**Conclusion:**

An individualised, item‐specific, nurse‐implemented OHC plan guided by structured screening and clinical assessment improved gingival inflammation and oral hygiene compared with routine care in adults with ABI within a multidisciplinary neurorehabilitation setting. Validation and feasibility testing in randomised clinical trials are warranted to facilitate translation into clinical practice.

## Graphical abstract

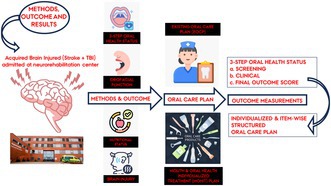

## 1 Introduction

Oral health is a fundamental component of systemic health, yet it remains an underrecognized element in hospital and rehabilitation medicine [1]. In individuals with acquired brain injury (ABI), maintaining adequate oral hygiene is particularly challenging due to impaired motor function, dysphagia, cognitive deficits, and dependence on caregivers for daily oral care [2, 3, 4, 5]. These factors often lead to plaque accumulation, gingival inflammation, halitosis, and secondary infections, collectively compromising oral comfort, nutrition, and quality of life [6, 7, 8, 9, 10, 11, 12]. Despite well‐established links between poor oral hygiene and systemic complications, including pneumonia and malnutrition, oral care remains a neglected area of clinical practice in medical and rehabilitation settings [6, 8, 13, 14, 15]. Epidemiological evidence shows that institutionalised and hospitalised populations, including ABI, exhibit poor oral health, with high prevalence of dental plaque, gingival bleeding, hyposalivation, dental caries, and periodontitis [13, 14, 16].

Several national and international guidelines have recognised oral health as an essential component of stroke and rehabilitation care [17, 18, 19, 20, 21]. Nevertheless, implementation remains poor due to limited training, unclear professional responsibilities, and competing clinical priorities among health‐care professionals [22, 23]. Although health professionals generally express a positive attitude toward oral health care (OHC) in hospitalised settings, a recent survey highlights inadequate continuing education, over‐burdened shoulders, and a lack of practical, evidence‐based protocols suited for complex neurological patients [24].

Over the past decade, our research group has systematically examined oral health concepts among hospitalised ABI individuals using a multifactorial approach that accounts for systemic complications. We identified severe baseline oral disease burden and progressive deterioration during hospitalisation, linked to poor socio‐behavioural profiles and reduced oral health–related quality of life [11, 13, 14, 25, 26]. Further studies demonstrated that poor oral hygiene, dysphagia, dependency on tube feeding, and orofacial motor dysfunction contribute to malnutrition and elevate the risk of pneumonia, a leading cause of death in ABI populations [6].

Existing oral care plans (EOCP) lack diagnostic structure and individualised guidance, resulting in variable outcomes and fragmented implementation [27]. Although few intervention studies have demonstrated that systematic oral care can improve oral hygiene and few claim to even reduce pneumonia risk in vulnerable populations, most evidence is constrained by small samples, inconsistent outcome measures, or poorly defined interventions [9, 10, 28, 29, 30]. Consequently, OHC practices across the care continuum remain heterogenous, contributing to delayed diagnosis and unfocused treatment strategies [11, 27, 31, 32].

To address these gaps, we developed the Mouth and Oral Health Individualised Treatment (MOHIT) plan, an evidence‐based, nurse‐implemented framework integrating structured oral diagnostics with individualised OHC delivery. The MOHIT plan is guided by a two‐step diagnostic model that generates a Final Outcome Score (FOS) to classify patients by dependency level and oral health status. Based on this assessment, an item‐wise protocol targets specific oral structures (teeth, gingiva, tongue, mucosa, lips, and saliva), with individualised instructions for both dependent and independent patients. The plan bridges the gap between dental expertise and health care professionals (nursing) implementation through a systematic, interdisciplinary approach.

Therefore, the present cluster nonrandomized controlled trial aimed to evaluate the effectiveness of the individualised MOHIT plan compared with the EOCP plan in adults with ABI during inpatient rehabilitation. The hypothesis tested was that the MOHIT plan, guided by a structured two‐step diagnostic model, would yield greater reductions in gingival inflammation (bleeding on probing) and plaque accumulation, along with improved overall oral health scores, compared with routine oral care.

## 2 Material and Methods

### 2.1 Study Design, Participants and Recruitment

This cluster nonrandomized controlled trial was conducted at the Hammel Neurorehabilitation Center and University Research Clinic (HNRC) between February 2023 and September 2023. Individuals aged > 18 years with acquired brain injury (ABI), either from trauma (head injury, physical trauma due to accident and assault, neurosurgery, etc.) or non‐traumatic injury (stroke, brain tumour, infection, hypoxia, and anoxia, etc.) were recruited. Individuals admitted for any other reasons than ABI, such as neurodegenerative disorders and polyneuropathy, were excluded. Pregnant women were also excluded, as hormonal changes that occur during pregnancy might influence the oral and orofacial health assessment [33].

**FIGURE 1:**
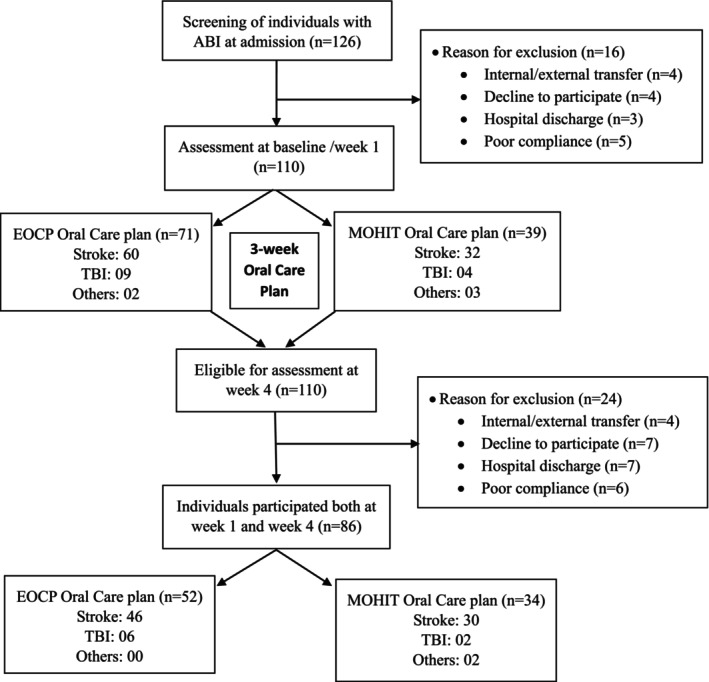
Flow‐chart. ABI, Acquired Brain Injury; EOCP, Existing Oral Care Plan; MOHIT, Mouth and Oral Health Individualised Treatment plan: TBI, Traumatic Brain Injury.

A total of 126 individuals were screened within the first week of admission (baseline) and reassessed at Week 4 to evaluate oral health changes (Figure 1). Of these, 110 completed the Week‐1 assessment and 86 completed Week 4 follow‐up. Some patients moved in and out of HNRC due to medical emergencies. In case those patients were re‐admitted within 5 days from their first day of admission at HNRC, they were included in the study [11]. The readmission day was counted as their first day of admission. Participants unable to complete the oral examination due to fatigue, limited mouth opening, stress, or acute infection were rescheduled within 1 week and if it was still not possible to re‐examine within a 1‐week window, they were excluded from the study (Figure 1).

#### 2.1.1 Cluster Allocation

**FIGURE 2:**
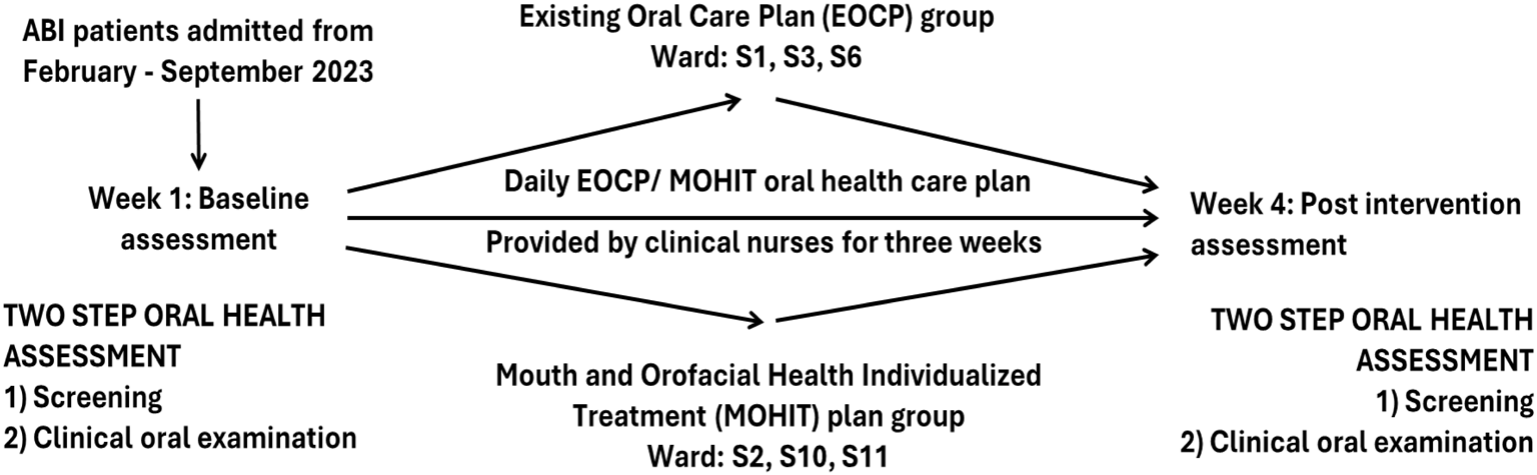
Study design.

HNRC comprises eight specialised neurorehabilitation wards for individuals with ABI. Prior to trial initiation, ward leaders and oral‐care representatives were briefed on the study rationale, intervention procedures, and logistical requirements. As oral care practices cannot be blinded and nursing staff rotate within wards, individual‐level randomization was not feasible; therefore, ward‐level (cluster) allocation was required to maintain intervention fidelity and minimise contamination between study arms. Clusters were assigned pragmatically based on clinical capacity, staffing stability, and willingness to participate, reflecting real‐world neurorehabilitation workflows and minimising disruption to routine care. Wards S1, S3, and S6 continued with the EOCP, whereas wards S2, S10, and S11 implemented the MOHIT plan. One ward (S5) declined participation due to staffing shortages and concurrent research commitments. The final cluster configuration is illustrated in Figure 2. Nurses in MOHIT wards received targeted training in the intervention protocol prior to implementation, while EOCP wards continued standard practice without additional instruction. Although baseline characteristics were broadly comparable between groups, the nonrandomized cluster design carries an inherent risk of residual confounding, which was addressed analytically using mixed‐effects models incorporating ward‐level random intercepts.

The project was notified to the Central Denmark Region Committee on Health Research Ethics (case no. 1‐10‐72‐124‐22). In accordance with the Danish Consolidation Act on Research Ethics Review of Health Research Projects (Act no. 1338 of 1 September 2020, section 14 (1)), the committee classified it as a “clinical quality development project,” exempting it from formal ethical approval and written informed consent. Institutional review board approval was obtained (case no. 196525). The study complied with the Helsinki Declaration II; participants received verbal and written information and were free to decline participation at any time.

#### 2.1.2 Sample Size Estimation

Sample size estimation was based on detecting a 20% absolute difference in oral health improvement between groups. We expected reductions in bleeding on probing (BOP) and plaque levels of 15% in the EOCP group and 35% in the MOHIT group. Power was set at 80% with a two‐sided significance level (α) of 0.05. Based on these assumptions, a minimum of 50 participants per group was required. Allowing for a 10% attrition rate, the total target sample size was 110 participants. The initial sample size calculation did not incorporate an intracluster correlation coefficient (ICC) or design effect, as reliable ward‐level ICC estimates for oral health outcomes in individuals with ABI were unavailable at the time of study planning. To account for clustering and repeated measures, all primary and secondary analyses were therefore conducted using mixed‐effects modelling frameworks with random effects at the ward and participant levels. This analytical strategy mitigates the impact of within‐cluster correlation on effect estimation.

### 2.2 Demographics, Medical and Socio‐Behavioural History

Demographic and behavioural information was collected by the project nurse using a structured questionnaire at both baseline (Week 1) and follow‐up (Week 4). Variables included age, sex, education level (basic, vocational, high school, or higher education), smoking status, and oral‐hygiene habits such as toothbrush type (manual, electric, or both), brushing frequency, and dental‐visit regularity prior to hospitalisation. In addition, changes in brushing frequency and toothbrush type during hospitalisation were also recorded at Week 4.

Medical information, including primary diagnosis, comorbidities (e.g., diabetes, hypertension), brain‐injury aetiology, and length of stay in acute care, was extracted from electronic medical records. As part of standard neurorehabilitation procedures, data on dysphagia, feeding status, eating difficulties, pneumonia occurrence, and body‐mass index were collected by certified health professionals at both points to monitor recovery progress. The case definition for pneumonia was diagnosed based on antibiotic treatment. The complete list of variables was documented at baseline and Week 4 and presented in Table 1.

**TABLE 1:** Demographic and baseline characteristics by intervention group (MOHIT V/S EOCP).

Demographics and characteristics	Week 1 N (%)		Week 4 N (%)	
	EOCP	MOHIT	EOCP	MOHIT
Age, mean (SD)	57.7 (14.2)	55.7 (15.8)	—	—
Sex				
Male	50 (64.1%)	28 (35.9%)	—	—
Female	21 (65.6%)	11 (34.4%)	—	—
BMI, mean (SD)	26.0 (4.8)	26.0 (4.6)	—	—
Diagnosis				
Stroke	60 (65.2%)	32 (34.8%)	—	—
TBI	9 (69.2%)	4 (30.8%)	—	—
Others	2 (40.0%)	3 (60.0%)	—	—
Length of stay in acute care (days), mean (SD)	19.2 (15.8)	14.1 (11.3)	—	—
Onset of brain injury (days), mean (SD)	39.5 (32.8)	30.9 (22.9)	—	—
Feeding status				
Oral	55 (67.1%)	27 (32.9%)	44 (59.5%)	30 (40.5%)
Nasal	8 (50.0%)	8 (50.0%)	1 (50.0%)	1 (50.0%)
PEG	8 (66.7%)	4 (33.3%)	8 (57.1%)	6 (42.9%)
Dysphagia				
Yes	32 (72.7%)	12 (27.3%)	20 (71.4%)	8 (28.6%)
No	37 (57.8%)	27 (42.2%)	32 (52.5%)	29 (47.5%)
Eating difficulties				
Yes	15 (68.2%)	7 (31.8%)	3 (42.9%)	4 (57.1%)
No	44 (62.0%)	27 (38.0%)	44 (59.5%)	30 (40.5%)
Pneumonia up to 20 days before admission				
Yes	3 (60.0%)	2 (40.0%)	—	
No	68 (64.8%)	37 (35.2%)	—	
Pneumonia during hospitalisation				
Yes	—		2 (100%)	
No	—		56 (59.6%)	38 (40.4%)
Diabetes				
Yes	13 (68.4%)	6 (31.6%)	—	—
No	58 (63.7%)	33 (36.3%)	—	—
Hypertension				
Yes	22 (59.5%)	15 (40.5%)	—	—
No	45 (40.9)		—	—
Smoking				
Never	22 (59.5%)	15 (40.5%)	—	—
Former	28 (60.9%)	18 (39.1%)	—	—
Current	11 (73.3%)	4 (26.7%)	—	—
Toothbrushing frequency/daily				
Less than once	1 (50.0%)	1 (50.0%)	—	—
1	9 (64.3%)	5 (35.7%)	10 (66.7%)	5 (33.3%)
2	40 (60.6%)	26 (39.4%)	24 (51.1%)	23 (48.9%)
3 or more	—	2 (100.0%)	2 (28.6%)	5 (71.4%)
Type of toothbrush				
None	1 (100%)	—	—	—
Manual	23 (54.8%)	19 (45.2%)	25 (78.1%)	7 (21.9%)
Electric	18 (64.3%)	10 (35.7%)	8 (26.7%)	22 (73.3%)
Both	7 (58.3%)	5 (41.7%)	3 (50.0%)	3 (50.0%)
Dental visits in the past 12 months				
None	14 (70.0%)	6 (30.0%)	—	—
1	14 (53.8%)	12 (46.2%)	—	—
2	17 (65.4%)	9 (34.6%)	—	—
3	1 (33.3%)	2 (66.7%)	—	—
4	2 (50.0%)	2 (50.0%)	—	—
Seldom	1 (100.0%)	—	—	—
Caries, mean (SD)	0.5 (1.2)	0.6 (1.3)	—	—
Missing teeth, mean (SD)	2.2 (4.0)	1.9 (2.6)	—	—
Restoration, mean (SD)	3.7 (3.2)	2.9 (3.0)	—	—
Root stumps, mean (SD)	0.9 (2.7)	0.3 (1.3)	—	—
Fixed prosthesis, mean (SD)	0.8 (2.0)	0.7 (1.5)	—	—
Sites with Bleeding on probe, mean (SD)	66.3 (22.8)	63.4 (21.6)	67.7 (21.6)	46.2 (20.3)
Sites with PPD ≥ 3 mm, mean (SD)	34.0 (6.5)	34.9 (3.3)	34.6 (5.2)	34.8 (3.4)
Sites with PPD ≥ 4 mm, mean (SD)	29.2 (10.6)	27.5 (8.6)	30.2 (9.7)	26.9 (8.6)
Sites with PPD ≥ 5 mm, mean (SD)	26.1 (11.2)	23.7 (9.5)	25.7 (11.2)	23.5 (10.6)
Sites with PPD ≥ 6 mm, mean (SD)	20.5 (12.5)	16.3 (12.7)	19.2 (12.7)	16.1 (13.1)

Abbreviations: BMI, Body Mass Index; EOCP, Existing Oral Care Plan; MOHIT, Mouth and Oral Health Individualised Treatment; PEG, Percutaneous endoscopic gastrostomy; PPD, Periodontal Pocket Depth; SD, Standard Deviation; TBI, Traumatic Brain Injury.

### 2.3 Comprehensive Oral Health Assessment

A two‐step diagnostic protocol, including screening and clinical examination was performed by two calibrated dentists.

#### 2.3.1 Step 1: Screening by Modified Bedside Oral Examination (mBOE)

The modified Bedside Oral Examination (mBOE), adapted from the original BOE [34] and later modified and validated for ABI population was used as a screening tool [31] with the help of wooden spatula and torch by a trained dentist. The mBOE screening tool assessed (a) oral health components (tooth‐plaque, tooth‐caries, tongue, gingiva, odour, saliva, mucous membrane and lips) and (b) orofacial health components (swallowing, jaw movements, presence/absence of nasogastric tube or tracheostomy and care‐giver dependence for OHC). Each parameter was rated on a 3‐point scale, with higher scores indicating poorer condition (Table 2).

**TABLE 2:** Final outcome scores (FOS): combination of mBOE and clinical assessment.

Category	Methods of measurement	Normal function (1)	Moderate dysfunction (2)	Severe dysfunction (3)
1a. Teeth (plaque)	Plaque & calculus examination	No visible plaque (0%–33% of sites)	Mild to moderate plaque (34%–66% site)	Severe plaque/calculus (> 66%)
1b. Teeth (caries)	DMF‐T indexing	No caries	1–2 tooth carious sites	> 2 carious tooth sites
2. Gingiva	Bleeding on probing	No bleeding (0%–33% of sites)	Mild–moderate bleeding (34%–66%)	Severe bleeding (> 66%)
3. Tongue	Tongue coating index	0%–33% coated tongue	34%–66% coated tongue	> 66% coated tongue
4. Odour	Organoleptic measures	No bad breath	Slight to moderate foul odour	Strong foul odour
5. Saliva: Hyper/Hypo salivation	Unstimulated cotton roll method	Normal flow rate (0.3–0.5 mL/min)	Reduced flow rate (0.1–0.299 mL/min)	Severely reduced (< 0.1 mL/min)
		Normal flow rate (0.3–0.5 mL/min)	Excess flow rate (0.5–1.0 mL/min)	Hyper flow rate (> 1.0 mL/min)
6. Mucous membrane	Observe tissue appearance	Pink & moist	Coating ± redness	Ulcerated ± bleeding
7. Lips	Observe lip appearance	Pink & moist	Dry &/or cracked	Ulcerated ± bleeding
8. Oral candidiasis	Tongue clinical examination	No white lesions	Few white lesions with redness underneath	Many white patches ± pain/bleeding when scrapped

Abbreviation: DMF‐T, Decayed, Missing, Filled teeth indexing.

#### 2.3.2 Step 2: Clinical Oral Examination

*Full‐mouth clinical examination* was performed using a UNC‐15 periodontal probe (PCPUNC15, Hu‐Friedy, Chicago, IL, USA) at six sites per index tooth (16/15, 26/25, 36/35, 46/45, 11/21, 41/31). Parameters recorded included bleeding on probing (BOP), plaque, calculus, and periodontal probing depth (PPD). Plaque and calculus were assessed by gently passing the probe ≤ 1 mm below the gingival margin and graded according to standard criteria (Table 2).

*DMF‐T (Decayed, Missing, Filled teeth)‐index*: Presence of decayed (D), missing (M) and filled (F) teeth was assessed using the DMF‐T index with dental arch explorer and mirror to check the development of dental caries and the deterioration of oral hygiene. The presence of decayed teeth was a clinical indicator of caries activity (Table 2).

*Tongue coating index*: Tongue coating was evaluated utilising the criteria of Shimizu et al. [35]. The dorsal surface of tongue was into nine equal parts and depending on the coating, it was graded as normal, moderate or severe coating (Table 2).

Organoleptic assessment: Oral malodor was evaluated organoleptically by a trained examiner positioned 5–10 cm from the patient's mouth after 1 min of closed‐mouth breath retention [36]. The strength of the odour was rated according to the six‐point scale and was later rearranged to three categories as mentioned in Table 2.

*Unstimulated saliva examination*: Three sterile cotton rolls were placed intra‐orally (bilaterally between molars and one sublingual) for 2 min. The salivary volume was calculated from the pre‐ and post‐weights of the cotton rolls (1 g = 1 mL) [37] while the salivary flow rate was determined by dividing the volume of saliva collected by the time taken for the saliva collection (Table 2).

*Suspected oral candidiasis (Oral Thrush)*: Using a tongue blade or dental explorer under torchlight, trained dentists examined the tongue and oral mucosa for creamy white, slightly raised plaques with a cottage cheese–like appearance. Lesions that were removable on gentle scraping and revealed erythema, bleeding, or soreness underneath were classified as suspected oral candidiasis (Table 2).

### 2.4 Final Outcome Scores (FOS)

Composite scores from the screening and clinical examinations were integrated to yield a Final Outcome Score (FOS). Individuals were categorised as:

- 1 = normal function (≤ 33% of sites affected),
- 2 = moderate dysfunction (34%–66%), or
- 3 = severe dysfunction (> 66%).

Each parameter was rated on a 3‐point scale, with higher scores indicating poorer condition (Table 2). The FOS guided individualization of oral‐care protocols within the MOHIT plan.

### 2.5 Intervention

#### 2.5.1 Existing Oral Care Plan (EOCP)

The EOCP followed Denmark's national clinical guidelines for oral care [27] and represented standard hospital practice at HNRC. Oral hygiene procedures were delivered using conventional toothbrushes and toothpaste, supplemented by additional care as required. No protocol modifications were made for study purposes; however, all outcome assessments mirrored those of the MOHIT group. The details of EOCP are explained in Table 3A.

**TABLE 3A:** Existing oral care plan (EOCP).

Individuals at HNRC	Standard oral care (recommended clinical guidelines)	Supplemental oral care (case‐dependent)
Patients with self‐oral care	Instruction to brush twice daily, preferably after each meal Free to have any toothbrush they bring from home (small head/big head/electric soft bristle toothbrush) with fluoride toothpaste (1450 ppm)	Chlorhexidine mouth wash (0.12%) Oral mucosal care Dental floss once a day Lip moisturiser for dry or cracked lips
Patients with swallowing/eating/motor/cognitive difficulties (oral care by caregivers)	Cleaning of mouth for food debris and secretions before and after each intake of food and drinks Use of small head soft bristle toothbrush and fluoridated non‐foaming toothpaste (1450 ppm) Tooth brushing in circulatory motion starting bucally, palatally and then to occlusal table twice a day after meal	Chlorhexidine mouth wash (0.12%) Oral mucosal care Lip moisturiser for dry or cracked lips

Abbreviation: HNRC, Hammel Neuro Rehabilitation Center and University Research Clinic.

#### 2.5.2 MOHIT Plan

The MOHIT plan was derived from FOS, where each individual received a tailored scoring system for each category ranging from 1 to 3 depending on their oral health status (Table 2), which help produce an individualised, item‐wise OHC plan targeting specific domains (tooth‐plaque, tooth‐caries, tongue, gingiva, odour, saliva, mucosa, lips). OHC delivery was stratified by patient dependency (self‐care vs. caregiver‐assisted). Each participant received oral care instructions verbally by the nurses and were also posted bedside for the 3‐week intervention period, and nurses received structured training and supervision from a dental researcher. Oral care products were supplied to each patient's room as mentioned in Table 3B. All the patients under MOHIT plan were recommended to sit as upright as possible during OHC delivery to prevent any aspiration. Even patients with healthy mouths were provided with a baseline OHC plan which consisted of the use of small, softheaded toothbrush with a smear of nonfoaming 1450 ppm fluoridated toothpaste twice a day for two minutes each time (2 × 2 rule) after meals. In addition, they were advised to use the tongue scrapper to clean the tongue once per day and apply water‐based lip moisturisers to keep their lips moist. Interdental brushes were also provided to remove food debris from in between the teeth (Table 3B).

**TABLE 3B:** MOHIT treatment plan.

Category	Individualised oral care plan (either after breakfast/lunch/dinner)	Normal function (1)	Moderate dysfunction (2)	Severe dysfunction (3)
1a. Teeth with plaque (Patients with self‐care)	(a) Paediatric toothbrush for 2 min (b) Interdental brush (if only patient eats by mouth)	XX^a^		
	(a) Electric sonic toothbrush for 2 min (b) Interdental brush (if only patient eats by mouth)		XX	XXX
1b. Teeth with plaque (Patients dependent on caregivers)	(a) Electric suction toothbrush 2 min (b) Interdental brush (if only patients eat by mouth)	XX	XX	XXX
1b. Teeth with caries	Fluoridated paste (1450 ppm)	XX	XX	XXX
2. Tongue	Tongue scrapper after tooth brushing (back to front)	X	XX	XXX
3a/4a/8a. Gingiva/Odour/Candidiasis (Patients with self‐care)	20 mL, 0.2% Chlorhexidine (CHX) mouth wash for 30 s in mouth, gargle & then spit (after tongue scraping)	—	X	XX
3b/4b/8b. Gingiva/Odour/Candidiasis (Patients dependent on caregivers)	Dip toothbrush into 20 mL, 0.2% CHX mouth wash and apply in the entire mouth covering tongue, tooth, gum for 30 s (after tongue scraping)	—	X	XX
5a/6. Dry mucosa/Dry mouth	Oral moisturiser/toothpaste with moisturiser	—	XX	XXX
5b. Hypersalivation	Use of suction tube/cotton rolls for excess saliva removal	—	XX	XXX
7. Lips	Water‐based moisturisers for lips	X	XX	XXX
9. Respiratory pathogens	Consult physician for probable pneumonia	—	—	—

Abbreviation: MOHIT, Mouth and Oral Health Individualised Treatment plan.

^a^ X: one time/day.

For moderate to severe dysfunction category, frequency and intensity of care were increased proportionally to FOS severity (Table 3B). For example, coated tongues were cleaned 1–3 times daily using a tongue scraper, and patients with bleeding gums, oral malodor, or clinical candidiasis used 0.2% chlorhexidine (CHX) mouthwash (20 mL for 30 s) or CHX‐dipped toothbrush applications as needed. Dry‐mouth cases were managed with hydrated toothpaste and water‐based lip emollients (Table 3B).

Individuals requiring support from health professionals: For patients with dysphagia, cognitive, physical, or motor impairments, OHC was entirely performed by trained nurses. A suction electric toothbrush (Bluereo G100, South Korea) was used to facilitate toothbrushing. The built‐in LED light improved intraoral visibility, enhancing caregiver precision and confidence during OHC and the inbuilt suction helped to remove excess saliva from the mouth which possibly could have been aspirated to the lungs (Table 3B). In cases of gingival bleeding, oral malodor, or suspected candidiasis, nurses immersed the brush head in 20 mL of 0.2% chlorhexidine (CHX) solution and gently applied it to all oral surfaces, including teeth, tongue, and gingiva (Table 3B).

### 2.6 Analytical Approach

Descriptive statistics summarised baseline and Week‐4 data. Means with standard deviations were calculated for continuous variables, and absolute and relative frequencies for categorical variables. The primary outcome was the change in BOP from baseline to Week 4. Secondary outcomes included plaque index, tongue‐coating score, oral malodor, oral candidiasis, and mBOE total and sub‐scores.

Given the clustered and repeated‐measures design, mixed‐effects models were applied. For continuous outcomes (e.g., BOP, plaque, salivary flow rate), linear mixed‐effects models were used; for ordinal outcomes (e.g., tongue coating, mBOE and its sub‐scores), cumulative link mixed models with Laplace approximation were fitted. All models included fixed effects for group (MOHIT vs. EOCP), time (baseline vs. Week 4), and their interaction, and random intercepts for wards (S1–S11) and patient to account for clustering and repeated observations.

Models were adjusted for age, sex, baseline value of the outcome, and feeding status. Toothbrush type was treated as an ordinal variable (0 = none, 1 = manual, 2 = electric, 3 = both). The key parameter of interest was the group × time interaction, representing the differential change between MOHIT and EOCP. Results were expressed as adjusted mean differences or odds ratios with 95% confidence intervals. Model assumptions (normality, proportional odds, homoscedasticity) were verified. Missing data were handled by restricted maximum‐likelihood estimation, assuming data were missing at random. Sensitivity analyses included additional adjustments for dysphagia severity and length of stay. All tests were two‐sided with *p* < 0.05 considered significant. Analyses were performed in R (version 4.3.3).

## 3 Results

### 3.1 Descriptive Results

The participants had a mean age of 57.1 years (±14.8), with similar age distributions between groups (EOCP: 57.7 ± 14.2; MOHIT: 55.7 ± 15.8). Most participants were male. Stroke was the predominant diagnosis, accounting for the majority of cases in both groups, followed by traumatic brain injury, while other neurological conditions were less frequent. The mean length of stay in acute care was 17.5 days (±14.6), being longer in the EOCP group (19.2 ± 15.8 days) than in the MOHIT group (14.1 ± 11.3 days). The mean time from brain injury onset to admission was 36.2 days (±29.1), again slightly higher in EOCP (39.5 ± 32.8) compared with MOHIT (30.9 ± 22.9; Table 1).

At admission, most participants were orally fed (67.1% in EOCP and 32.9% in MOHIT), with smaller proportions using nasal feeding or PEG. Dysphagia was present in approximately 44% of participants at baseline, decreasing by Week 4, when 28 individuals (31.5%) still presented dysphagia. Similarly, eating difficulties were reported by 23.7% of participants at admission and declined substantially to 8.6% after 4 weeks, with a more pronounced relative reduction observed in the EOCP group (Table 1). Five participants had pneumonia within the 20 days prior to admission (three in EOCP and two in MOHIT). During hospitalisation, pneumonia developed in two participants, both allocated to the EOCP group, while no new cases were observed in MOHIT. In this study, pneumonia diagnosis was established based on the initiation of antibiotic treatment.

Oral health behaviours remained largely stable over time. Toothbrushing twice daily was the most common pattern at both time points (60.6% at Week 1 and 51.1% at Week 4), while the proportion of participants brushing three or more times per day increased at Week 4, particularly in the MOHIT group. The use of electric toothbrushes increased over time, with a marked shift toward electric brushing in the MOHIT group at week 4 (73.3%). Dental visits in the previous 12 months were heterogeneous, with most participants reporting between one and two visits (Table 1). Baseline dental status showed a low mean number of untreated caries (0.6 ± 1.3 teeth), alongside 2.1 ± 3.6 missing teeth, 3.3 ± 3.1 restored teeth, and 0.6 ± 2.2 root stumps. Approximately one quarter of participants used fixed prostheses. These indicators were comparable between groups at baseline (Table 1).

Periodontal parameters showed modest changes over the study period. Mean sites with bleeding on probing were 65.0 ± 22.4 at baseline and remained relatively stable by week 4, with divergent trends between groups (EOCP: slight increase; MOHIT: reduction). The distribution of periodontal probing depths demonstrated minimal variation over time. Mean sites with PPD ≥ 3 mm remained stable (34.3 ± 5.5 at baseline vs. 34.7 ± 4.7 at week 4), as did PPD ≥ 4 mm (28.5 ± 9.8 vs. 28.6 ± 9.3), PPD ≥ 5 mm (25.1 ± 10.5 vs. 24.6 ± 10.9), and PPD ≥ 6 mm (18.9 ± 12.6 vs. 17.8 ± 13.0; Table 1).

### 3.2 Intervention Effects

#### 3.2.1 Primary Outcome: Bleeding on Probing (BOP)

**FIGURE 3:**
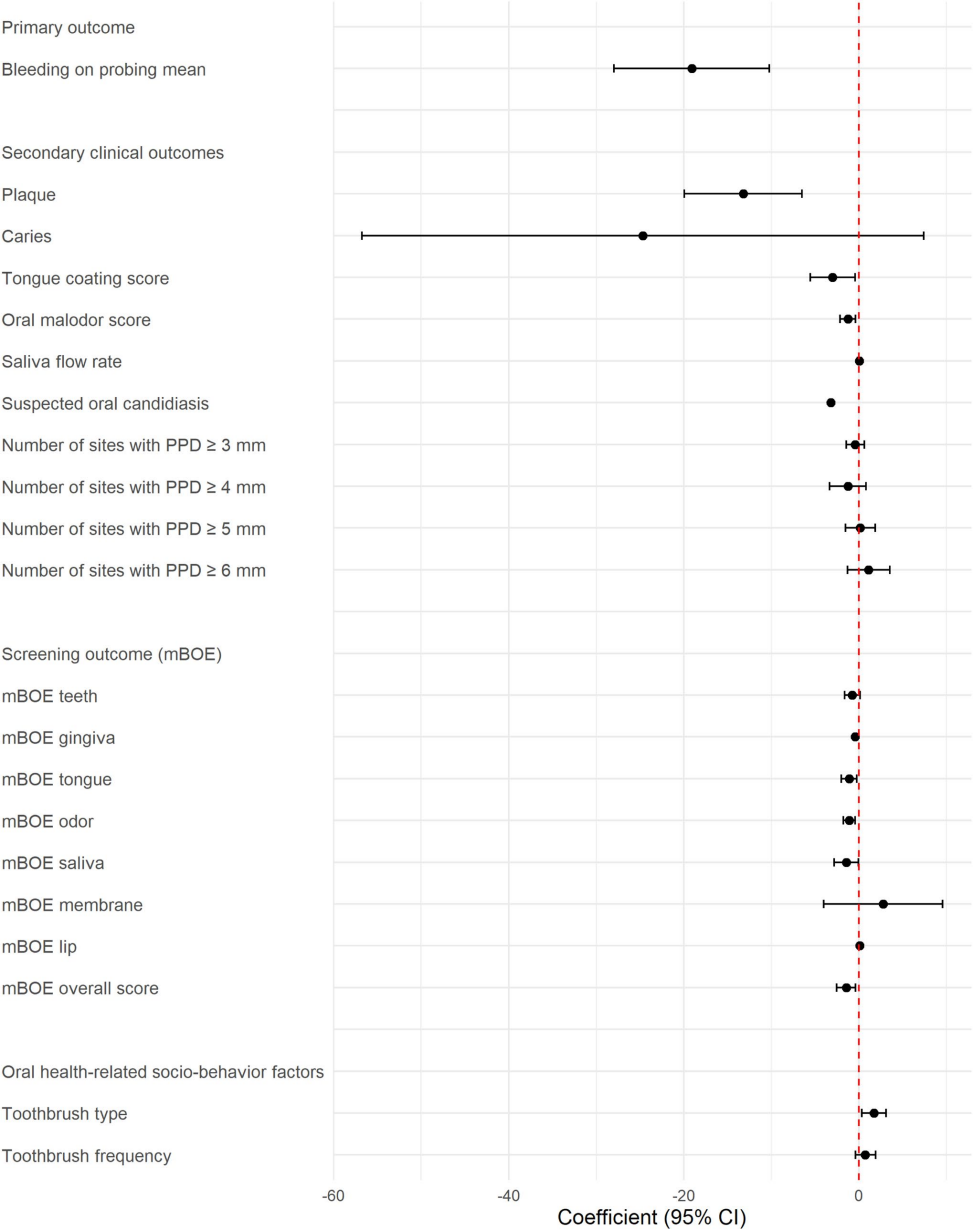
Comparison of the differences in oral measurements over time (baseline—Week 4) between the intervention groups. Coefficients and the 95% CIs were estimated using Linear Mixed‐Effects models for continuous variables and Cumulative Link Mixed Models for ordinal variables, both adjusted for age and sex. Comparison between EOCP (reference, 0.0) and MOHIT groups: An interval below zero indicates a decrease in the parameter over time in the MOHIT group compared to EOCP; an interval above zero indicates an increase; and if the interval includes zero, no significant difference was observed. EOCP group as reference (00.0). EOCP, Existing Oral Care Plan; MOHIT, Mouth and Oral Health Individualised Treatment plan; mBOE, modified Bedside Oral Examination; PPD, Periodontal Pocket Depth.

Between‐group analysis (Week 4 minus baseline) showed that MOHIT was associated with a greater reduction in BOP than EOCP (coeff: −19.11; 95% CI: −27.97 to −10.24) (Table 4; Figure 3). Within‐group change was significant in MOHIT (coeff: −18.04; 95% CI: −24.61 to −11.48; Table 5).

**TABLE 4:** Comparison of the difference in oral measurements over time between the intervention groups.

	Difference (Week 4—Baseline)		
	Coeff.	95% CI	*p*
*Primary outcome*			
Bleeding on probing mean	−19.11	−27.97; −10.24	< 0.001
*Secondary clinical outcomes*			
Plaque	−13.23	−19.93; −6.53	< 0.001
Caries	−24.68	−56.76; 7.40	0.992
Tongue coating score	−3.01	−5.58; −0.45	0.022
Oral malodor score	−1.27	−2.15; −0.40	0.004
Saliva flow rate	0.03	−0.16; 0.22	0.725
Suspected oral candidiasis	−3.21	−3.22; −3.20	< 0.001
Number of sites with PPD ≥ 3 mm	−0.43	−1.47; 0.60	0.408
Number of sites with PPD ≥ 4 mm	−1.28	−3.34; 0.78	0.219
Number of sites with PPD ≥ 5 mm	0.14	−1.55; 1.83	0.870
Number of sites with PPD ≥ 6 mm	1.09	−1.32; 3.50	0.369
*Screening outcomes (mBOE)*			
mBOE teeth	−0.78	−1.66; 0.11	0.084
mBOE gingiva	−0.44	−0.45; −0.46	< 0.001
mBOE tongue	−1.11	−2.00; −0.26	0.012
mBOE odour	−1.12	−1.78; −0.46	0.001
mBOE saliva	−1.44	−2.84; −0.04	0.044
mBOE membrane	2.74	−4.05; 9.53	0.430
mBOE lip	0.08	0.09; 0.10	< 0.001
mBOE overall score	−1.46	−2.54; −0.39	0.008
*Oral health‐related socio‐behaviour factors*			
Toothbrush type	1.70	0.33; 3.07	0.015
Toothbrush frequency	0.70	−0.41; 1.89	0.225

*Note:* Coefficients were estimated using Linear Mixed‐Effects models for continuous variables and Cumulative Link Mixed Models for ordinal variables, both adjusted for age and sex, with a significance level set at 5%. EOCP group as reference (00.0).

Abbreviations: EOCP, Existing Oral Care Plan; mBOE, modified Bedside Oral Examination; MOHIT, Mouth and Oral Health Individualised Treatment plan; PPD, Periodontal Pocket Depth.

**TABLE 5:** Comparison of oral measurements over time within each intervention group.

	EOCP			MOHIT		
	Coeff.	95% CI	*p*	Coeff.	95% CI	*p*
*Primary outcome*						
Bleeding on probing mean	0.78	−4.81; 6.37	0.780	−18.04	−24.61; −11.48	< 0.001
*Secondary clinical outcomes*						
Plaque	−4.92	−9.36; −0.48	0.031	−17.88	−22.43; −13.33	< 0.001
Caries	0.08	−0.64; 0.80	0.837	−0.10	−0.46; 0.66	0.094
Tongue coating score	−1.59	−3.35; 0.16	0.074	−4.71	−6.07; −3.35	< 0.001
Oral malodor score	−0.30	−0.31; −0.30	< 0.001	−2.97	−4.58; −1.37	< 0.001
Saliva flow rate	−0.08	−0.19; 0.02	0.120	−0.07	−0.22; 0.07	0.317
Suspected oral candidiasis	−20.87	−20.87; −20.87	< 0.001	−22.39	−23.46; −21.32	< 0.001
Number of sites with PPD ≥ 3 mm	−7.68	−11.24; −4.11	< 0.001	−3.72	−7.3; −0.13	0.042
Number of sites with PPD ≥ 4 mm	−6.25	−9.49; −3.02	< 0.001	−3.28	−6.51; −0.06	0.046
Number of sites with PPD ≥ 5 mm	−6.51	−9.44; −3.58	< 0.001	−2.59	−5.02; −0.16	0.037
Number of sites with PPD ≥ 6 mm	−5.8	−8.45; −3.16	< 0.001	−1.87	−3.67; −0.07	0.042
*Screening outcome (mBOE)*						
mBOE teeth	−0.41	−1.31; 0.49	0.370	−2.49	−4.56; −0.42	0.018
mBOE gingiva	−2.88	−5.25; −0.51	0.017	−1.96	−14.91; −14.89	< 0.001
mBOE tongue	−0.28	−0.99; 0.43	0.434	−2.83	−4.46; −1.21	0.001
mBOE odour	−0.35	−1.14; 0.43	0.378	−3.68	−6.19; −1.17	0.004
mBOE saliva	−0.28	−1.20; 0.64	0.548	−2.62	−4.35; −0.89	0.003
mBOE membrane	−1.95	−4.12; 0.23	0.079	1.84	−2.32; 6.00	0.387
mBOE lip	−1.66	−1.67; −1.65	< 0.001	−2.21	−4.39; −0.03	0.047
mBOE overall score	−0.84	−1.58; −0.11	0.025	−2.35	−3.08; −1.63	< 0.001
*Oral health‐related socio‐behaviour factors*						
Toothbrush type	−1.31	−2.55; −0.08	0.037	1.18	0.04; 2.32	0.042
Toothbrush frequency	−0.74	−0.75; −0.73	< 0.001	1.66	−0.50; 3.83	0.133

*Note:* Coefficients were estimated using Linear Mixed‐Effects models for continuous variables and Cumulative Link Mixed Models for ordinal variables, both adjusted for age and sex, with a significance level set at 5%. Baseline as reference (00.0).

Abbreviations: EOCP, Existing Oral Care Plan; mBOE, modified bedside oral examination; MOHIT, Mouth and Oral Health Individualised Treatment plan; PPD, Periodontal Pocket Depth.

#### 3.2.2 Secondary Clinical Outcomes

Plaque, malodor and suspected oral candidiasis reduced in both the groups, but larger improvements were observed in the MOHIT group. Within MOHIT group, tongue coating scores (coeff: −4.71; 95% CI: −6.07 to −3.35) and oral malodor (coeff: −2.97; 95% CI: −4.58; −1.37) decreased exclusively (Table 5). Between groups, MOHIT outperformed EOCP on mean plaque (coeff: −13.23; 95% CI: −19.93 to −6.53), tongue coating (coeff: −3.01; 95% CI: −5.58 to −0.45), oral malodor (coeff: −1.27; 95% CI: −2.15 to −0.40) and suspected oral candidiasis (coeff: −3.21; 95% CI: −3.22 to −3.20; Table 4 and Figure 3). No statistical difference was observed in the number of decayed teeth in both groups (*p* > 0.094).

#### 3.2.3 Screening Outcomes (mBOE)

Within group comparisons, mBOE scores improved for teeth (coeff: −2.49; 95% CI: −4.56 to −0.42), tongue (coeff: −2.83; 95% CI: −4.46 to −1.21), oral malodor (coeff: −3.68; 95% CI: −6.19 to −1.17), and saliva (coeff: −2.62; 95% CI: −4.35 to −0.89) only in the MOHIT group. Overall mBOE scores, gingiva, and lip score improved in both groups, but with greater improvements in the MOHIT group (Table 5). Table 4 and Figure 3 displaying the comparisons between the intervention groups showed a significant improvement in the MOHIT group compared to the EOCP group for mBOE gingiva (coeff: −0.44; 95% CI: −0.46 to −0.45), tongue (coeff: −1.11; 95% CI: −2.00 to −0.26), odour (coeff: −1.12; 95% CI: −1.78 to −0.46), saliva (coeff: −1.44; 95% CI: −2.84 to −0.04), lip (coeff: 0.09; 95% CI: 0.08 to 0.10), and the overall BOE score (coeff: −1.46; 95% CI: −2.54 to −0.39).

#### 3.2.4 Oral Health‐Related Socio‐Behaviour Factors

Within‐group, MOHIT participants shifted toward electric or combined manual‐electric brushing (coeff: 1.18; 95% CI: 0.04 to 2.32), whereas EOCP shifted in the opposite direction (coeff: −1.31; 95% CI: −2.55 to −0.08; Table 4). Toothbrushing frequency decreased in EOCP group (coeff: −0.74; 95% CI: −0.75 to −0.73) and was unchanged in MOHIT group (coeff: 1.66; 95% CI: −0.50 to 3.83). Between groups, MOHIT demonstrated a greater shift toward more sophisticated toothbrush types (coeff: 1.70; 95% CI: 0.33 to 3.07) with no change in their toothbrushing frequency (coeff: 0.70; 95% CI: −0.41 to 1.89; Table 5 and Figure 3).

## 4 Discussion

This cluster‐controlled clinical trial evaluated the effectiveness of the MOHIT plan compared with the EOCP among adults with ABI during inpatient neurorehabilitation. The findings confirm the study hypothesis that the individualised, item‐specific, and dependency‐adapted MOHIT plan led to significant improvement on multiple oral health parameters, most notably gingival inflammation measured as bleeding on probing (BOP), compared with standard care (EOCP). Improvements were also observed in plaque accumulation, tongue coating, oral malodor, and suspected oral candidiasis. While modest gains in few parameters were seen in the EOCP group, which likely reflects the continued nursing efforts [27], the MOHIT plan produced broader and clinically meaningful effects within three weeks. These findings indicate that a structured, stepwise oral diagnostic and treatment framework can deliver tangible oral health gains in a complex neurological population. Given the nonrandomised cluster‐controlled design, these findings should be interpreted as evidence of association rather than causality.

Previous clinical studies on OHC for hospitalised populations have predominantly focused on screening tools, or generic care guidelines, which are useful for identifying risk but often lack diagnostic specificity, validation, and practical integration into nursing workflows [21, 31, 34, 38, 39]. Most existing oral care protocols also fail to differentiate care based on systemic, cognitive, or motor limitations, resulting in one‐size‐fits‐all approaches that underperform in dependent populations [6, 40]. In contrast, The MOHIT plan advances current practice by combining a validated oral diagnostic framework with individualised, item‐wise treatment steps and caregiver‐adapted instructions. This represents a shift from routine oral hygiene to precision‐based, function‐oriented oral care.

Among the most clinically relevant findings, the significant reduction in gingival inflammation (BOP) and plaque scores in the MOHIT group indicates effective disruption of bacterial biofilm and improved control of oral inflammation. Such outcomes are particularly meaningful in individuals with ABI, where inflammatory oral lesions can act as reservoirs for respiratory pathogens, contributing to aspiration pneumonia and other systemic inflammatory burden [6, 41, 42, 43, 44, 45, 46, 47]. The current results suggest that even modest improvements in oral hygiene can have far‐reaching systemic implications in this medically fragile group. Within this context, a distinctive feature of the MOHIT plan was the use of a suction electric toothbrush (Bluereo G100, South Korea) for patients' dependent on caregiver‐assisted oral care. Its LED illumination and sonic vibration improved visibility and plaque removal, while the built‐in suction efficiently cleared excess saliva and debris during brushing. These integrated features likely enhanced the removal of bacterial biofilm, tongue coating, and gingival exudate, which are key microbial reservoirs implicated in respiratory infections [15, 48]. By facilitating decontamination and improving salivary clearance, the intervention might have lowered the microbial burden available for aspiration into the lower airways. Notably, in the current study, all pneumonia cases present at baseline resolved during the intervention, whereas three new cases developed only in the EOCP group. Although a direct causal link cannot be established, the pattern suggests that enhanced oral decontamination, better salivary management, and increased caregiver confidence may have collectively lowered the risk of aspiration‐related respiratory complications. These findings align with growing evidence linking oral microbial dysbiosis and chronic gingival inflammation to respiratory pathogen colonisation and pneumonia risk [46, 47, 49, 50]. Further randomised or microbiologically focused studies are warranted to confirm this association and to explore the preventive potential of suction‐assisted oral hygiene in reducing pneumonia risk.

Beyond short‐term inflammatory outcomes, the broader implications of this study relate to the chronic oral‐systemic vulnerabilities seen in the ABI population. It has been evident from the literature that both short‐ (e.g., hospitalisation) and long‐term oral health is compromised in the ABI population, and their systemic complications like motor‐cognitive disability, dysphagia and tube feeding further hinder the maintenance of oral hygiene [6, 11, 26, 27]. Poor oral health and ABI have been shown to share a common environmental, socio‐behavioural and biological background [6]. Additionally, recent microbiological studies have also confirmed that even brief discontinuation of oral hygiene leads to changes in oral microbial community profiles like developing cariogenic microbiological species and disharmonizing oral homeostasis [49, 50]. Therefore, evidence‐based and personalised OHC intervention was essential for this vulnerable group and the current study highlights that the novel MOHIT plan was able to improve the oral health status significantly in comparison to the EOCP plan.

Beyond its mechanical and microbiological effects, the MOHIT plan demonstrates the translational potential of empowering nursing staff through structured, evidence‐based oral care training. Caregivers in the MOHIT wards received targeted instruction, individualised patient protocols, and visual reminders posted in patient rooms. Even with brief training, nurses successfully implemented individualised protocols, leading to measurable clinical gains within three weeks. This emphasises that oral health improvement in neurorehabilitation is not solely dependent on specialised dental interventions but can be achieved through interdisciplinary coordination, consistent nursing implementation, and diagnostic guidance from oral health professionals. The implementation strategy was intentionally pragmatic, mirroring real hospital workflows rather than relying on intensive dental supervision. Nurses were trained through short, focused sessions emphasising risk recognition, oral assessment, and individualised protocol application. Patients capable of self‐care received verbal instruction and ongoing encouragement, while dependent individuals were assisted using structured checklists derived from the diagnostic tool. This approach aligns with prior studies showing that even short educational interventions led by dental professionals can substantially improve staff knowledge, attitudes, and clinical outcomes in stroke or geriatric populations [5]. Furthermore, it has been shown that the caregivers lack attitude, knowledge and practices for OHC [24]. Hence, the current study bridges a persistent gap between dental and medical disciplines and contributes to the foundation of an evidence‐based OHC plan by identifying barriers and facilitators.

The primary aim of this study was to evaluate the effectiveness of the new intervention plan. As a result, we did not assess the time required by caregivers or their opinions regarding the performance of the MOHIT plan. Future work should therefore examine caregiver workload, adherence, and perceived feasibility to identify operational constraints and optimise implementation fidelity. Evaluating the long‐term sustainability and scalability of the plan across diverse rehabilitation settings will also be critical, particularly given the shortage of dental professionals in hospital care. Economic evaluations are warranted to determine whether the cost of training and suction‐integrated equipment is justified by reductions in oral disease burden and potential downstream savings from prevented complications such as pneumonia. Ultimately, these next steps will determine how the MOHIT framework can evolve from a clinical innovation to a standardised element of hospital‐based rehabilitation care.

## 5 Limitations

The initial sample size was calculated to require at least 50 participants per group, including allowances for dropouts. However, recruitment within the predefined study period (February–Septmeber 2023) yielded 34 participants in the MOHIT group. To mitigate the potential impact of reduced sample size, a post hoc power analysis was performed using the observed difference in bleeding on probing (BOP) and an assumed paired correlation of 0.7. The analysis demonstrated a post hoc statistical power of 91%, indicating that the primary outcome remained adequately powered despite the smaller sample.

Another limitation concerns the evaluation of oral candidiasis. Although clinical indicators were documented, microbiological confirmation could not be performed in real time due to logistical constraints. Consequently, the study did not assess the effect of specific antifungal therapies. Instead, prophylactic use of chlorhexidine mouthwash was incorporated, which coincided with a significant reduction in clinically observed candidiasis. While this suggests a preventive benefit of improved oral hygiene and antimicrobial rinsing, definitive microbial validation would strengthen future studies.

Lastly, the intervention period was relatively short (three weeks), and long‐term sustainability or recurrence of oral conditions could not be assessed. Future research should include extended follow‐up, microbial sampling, and health‐economic analyses to determine whether the observed clinical benefits translate into reduced infection rates and overall healthcare costs.

## 6 Conclusion

In this cluster‐controlled study, an individualised, item‐specific, and dependency‐adapted oral health care (OHC) plan based on structured diagnostic assessment was associated with improved gingival inflammation, plaque control, and overall oral health among hospitalised adults with acquired brain injury. The MOHIT plan represents a pragmatic, evidence‐based approach that integrates dental diagnostics into routine nursing practice. Future studies should validate its long‐term feasibility, scalability, and cost‐effectiveness, particularly in resource‐limited hospital settings, to enable translation into standardised clinical care.

## Funding

M.K. received a grant from the Health Research Fund of the Central Denmark Region for the current study with grant number A3098. The authors' salary was supported by their respective institutions. Oral care products were donated by Bluereo (South Korea) and Sunstar (Switzerland). The funders and donors had no role in the study design, data collection, analysis, or interpretation or in the writing of the manuscript.

## Conflicts of Interest

The authors declare no conflicts of interest.

## Data Availability

The data in the current project are defined as sensitive personal data. The data cannot be shared publicly due to existing data protection laws in Denmark imposed by the Danish Data Protection Agency. The access may be granted on anonymized data and on a case‐by‐case basis after approval from the PI and the research group.
